# Annurca Apple Extract and Colorectal Cancer Prevention: Preliminary In Silico Evaluation of Chlorogenic Acid

**DOI:** 10.3390/diseases14010033

**Published:** 2026-01-14

**Authors:** Ludovico Abenavoli, Giuseppe Guido Maria Scarlata, Maria Luisa Gambardella, Domenico Morano, Nataša Milošević, Maja Milanović, Nataša Milić

**Affiliations:** 1 Department of Health Sciences, University “Magna Graecia”, 88100 Catanzaro, Italy; giuseppeguidomaria.scarlata@unicz.it (G.G.M.S.); marialuisa.gambardella@studenti.unicz.it (M.L.G.); domenico.morano1@studenti.unicz.it (D.M.); 2 Center for Chronic Liver Diseases, “Renato Dulbecco” University Hospital, 88100 Catanzaro, Italy; 3 Department of Pharmacy, Faculty of Medicine Novi Sad, University of Novi Sad, Hajduk Veljkova 3, 21000 Novi Sad, Serbia; natasa.milosevic@mf.uns.ac.rs (N.M.); maja.milanovic@mf.uns.ac.rs (M.M.); natasa.milic@mf.uns.ac.rs (N.M.)

**Keywords:** diet, health, prevention, antioxidants, pathogenesis

## Abstract

Background/Objectives: Colorectal cancer (CRC) is a leading cause of cancer morbidity and mortality worldwide. Despite therapeutic advances, prevention through dietary bioactives remains a promising strategy. The Annurca apple (*Malus pumila* Miller cv. Annurca), a Mediterranean food rich in chlorogenic acid, exhibits antioxidant and anti-inflammatory effects. This study evaluated, via molecular docking, the multi-target interaction profile of chlorogenic acid against key CRC-related proteins. Methods: The optimized 3D structure of chlorogenic acid was docked to ten protein targets implicated in CRC pathogenesis, using the GOLD v.2022.3.0 software. Validation of the docking protocol was achieved by re-docking native ligands (RMSD ≤ 2.0 Å). Binding affinities were assessed by ChemPLP scoring, and interaction networks were visualized in Maestro Schrödinger. Results: Chlorogenic acid displayed consistent binding across all evaluated targets (ChemPLP 57.12–69.66), showing the highest affinity for nAChR (69.66), CXCR2 (65.13), ERβ (63.18) and TGFBR2 (62.94). The ligand formed multiple hydrogen bonds and π-π stacking interactions involving Asp1040 (VEGFR-1), Cys919 (VEGFR-2), Lys320 (CXCR2), and Tyr195 residues (nAChR), contributing to strong complex stabilization. Interaction patterns in CYP19A1, ERβ, and ERRγ suggested potential modulation of hormonal and metabolic signaling. The compound also demonstrated stable binding to mTOR (60.01), indicating a possible inhibitory role in proliferative pathways. Collectively, these findings reveal a broad, polypharmacological binding profile involving angiogenic, inflammatory, and hormonal regulators. Conclusions: Chlorogenic acid acts as a promising multi-target ligand in CRC prevention, with our in silico evidence supporting its ability to modulate diverse oncogenic pathways. Further experimental studies are warranted to confirm its efficacy and translational potential.

## 1. Introduction

**Figure 1:**
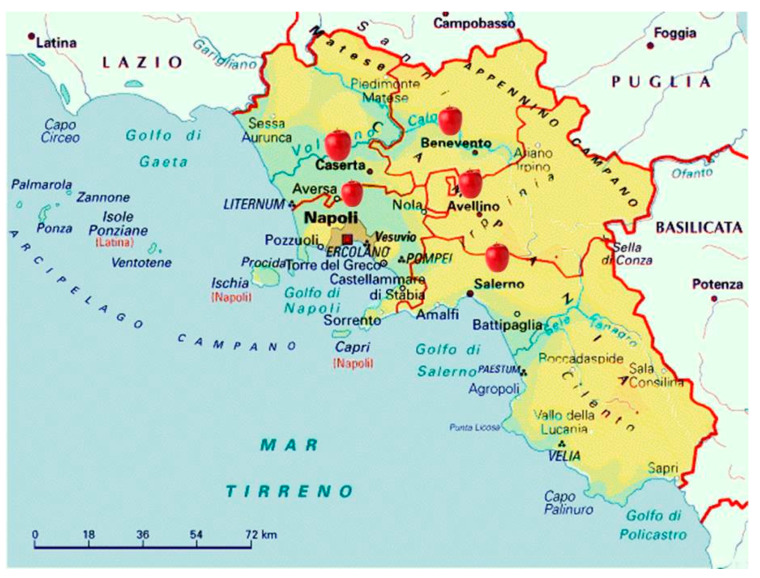
Geographical representation of PGI Annurca apple production, with a focus on the areas of Naples, Caserta, Avellino, Benevento, and Salerno (Campania Region, Southern Italy).

Colorectal cancer (CRC) is a major global health burden, ranking as the third most commonly diagnosed cancer and the second leading cause of cancer-related deaths worldwide [1]. According to the Global Cancer Observatory data from 2020, over 1.9 million new cases of CRC and approximately 935,000 deaths were recorded globally, with increasing incidence projected in both high-income and transitioning countries due to the adoption of Westernized lifestyles and dietary habits [2,3]. Despite advancements in screening and therapeutic modalities, the prognosis for CRC largely depends on the disease stage at diagnosis, thereby underscoring the importance of effective primary prevention strategies [4]. In this context, several epidemiological and experimental studies support the protective role of the traditional Mediterranean diet in the CRC prevention [5,6]. Characterized by high consumption of fruits, vegetables, legumes, whole grains, olive oil, and moderate red wine consumption, this dietary pattern is rich in polyphenols and bioactive phytochemicals with antioxidant, anti-inflammatory, and anti-proliferative properties [7]. Among the various components of the Mediterranean diet, fruit and, in particular, apples, have drawn attention due to their high content of polyphenolic compounds, including chlorogenic acid, quercetin, catechins, and procyanidins [8]. Specifically, the *Malus pumila* Miller cv. Annurca, a traditional apple cultivar native to Campania Region, Southern Italy (Figure 1), and recognized by the European Union as a Protected Geographical Indication (PGI) product, is characterized by a distinctive nutraceutical and phytochemical profile [9].

Unlike other apple varieties, Annurca apples undergo a traditional post-harvest reddening process that enhances their polyphenolic content [10]. Among its various phenolic constituents, chlorogenic acid, a major hydroxycinnamic acid, has emerged as a compound of considerable interest due to its multifaceted biological activities, including modulation of glucose and lipid metabolism, anti-inflammatory effects, and chemopreventive properties [11,12]. Furthermore, recent in vitro and in vivo studies have underscored the potential chemopreventive effects of Annurca apple polyphenols against CRC, demonstrating improvement in colonic damage in a rat model of colitis [13]. Moreover, regular apple consumption has been associated with a lower risk of CRC development [14]. In particular, in a recent meta-analysis, a higher apple consumption has been associated with a significant 25% reduction in CRC development [15]. Given these findings, the aim of the present study was to evaluate the interaction between chlorogenic acid and different molecular targets involved in CRC, based on a concomitant multiple-target approach.

## 2. Materials and Methods

### 2.1. Ligand Preparation

**Figure 2:**
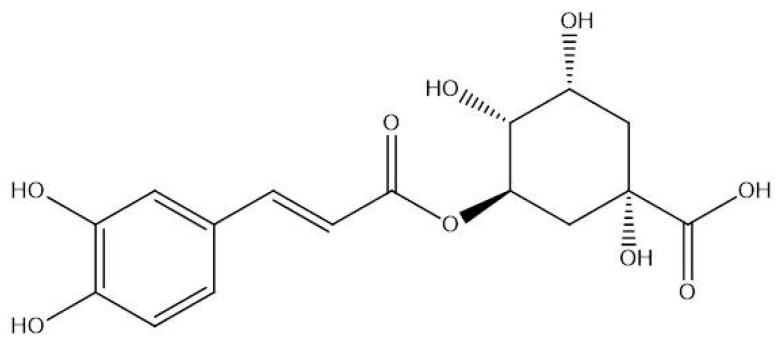
Chemical structure of chlorogenic acid.

The two-dimensional (2D) chemical structure of chlorogenic acid (Figure 2) was obtained from the PubChem database (https://pubchem.ncbi.nlm.nih.gov/, accessed on 12 February 2025) using its SMILES notation. The structure was redrawn using ChemDraw Professional, version 16.0, and the three-dimensional (3D) conformer was generated in Chem3D 16.0. Molecular geometry optimization was performed employing the MMFF94 force field to obtain the minimum-energy configuration suitable for docking analyses.

### 2.2. Protein Target Selection and Preparation

Ten molecular targets (Table 1) known to be implicated in colorectal carcinogenesis, angiogenesis, hormonal modulation, and cell signaling were selected through literature screening [16,17,18,19]. The crystallographic structures of the selected proteins were retrieved from the Protein Data Bank (PDB) (https://www.rcsb.org/). Targets and their corresponding PDB codes were included as follows: vascular endothelial growth factor receptor-1 (VEGFR-1, 3HNG), VEGFR-2 (4ASE), CXC chemokine receptor 2 (CXCR2, 6LFL), nicotinic acetylcholine receptor (nAChR, 4ZK4), transforming growth factor-β receptor type 2 (TGFBR2, 5QIN), aromatase (CYP19A1, 5JL6), mechanistic target of rapamycin (mTOR, 4JT5), estrogen receptor α (ERα, 7UJO), estrogen receptor β (ERβ, 5TOA), and estrogen-related receptor γ (ERRγ, 6KNR). Protein structures were prepared in the Hermes interface of the Genetic Optimization for Ligand Docking software (GOLD, v 2022.3.0) by removing co-crystallized ligands, ions, and water molecules. Each structure was protonated at physiological pH (7.4), and active sites were defined as spheres with a 6 Å radius centered on the coordinates of the native ligand.

### 2.3. Docking Protocol and Validation

Molecular docking was performed using the GOLD (v. 2022.3.0) on a Windows 10 64-bit platform. To validate the docking methodology, the native co-crystallized ligands for each target were re-docked into their corresponding binding sites, and root-mean-square deviation (RMSD) values between experimental and predicted poses were calculated. RMSD values below 2.0 Å confirmed the reliability and precision of the docking parameters. The ChemPLP scoring function was selected to evaluate binding affinity, as it provides a robust empirical estimate of protein–ligand interaction energy. Ligand flexibility was fully enabled, while protein residues within the binding cavity were kept rigid. The highest ChemPLP value was used for the discussion of the obtained results because it indicates the best ligand-target pose [20].

### 2.4. Visualization and Interaction Analysis

**Figure 3:**
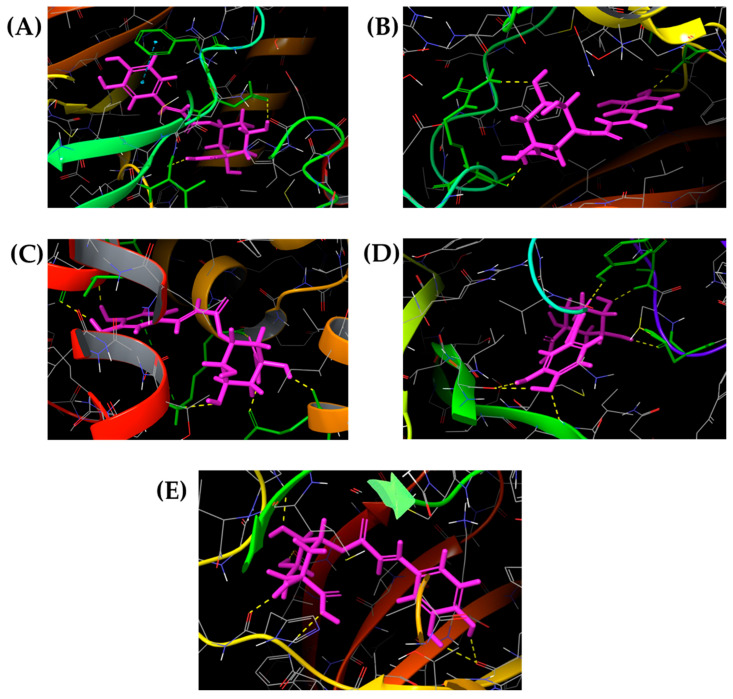
Three-dimensional representation of chlorogenic acid docked with (**A**) VEGFR-1, (**B**) VEGFR-2, (**C**) CXCR2, (**D**) nAChR, and (**E**) TGFBR2. Yellow dashes were used to mark hydrogen bonds while there are blue dashes for π-π interaction. Chlorogenic acid is marked in purple color while important amino acid residues are in green.

**Figure 4:**
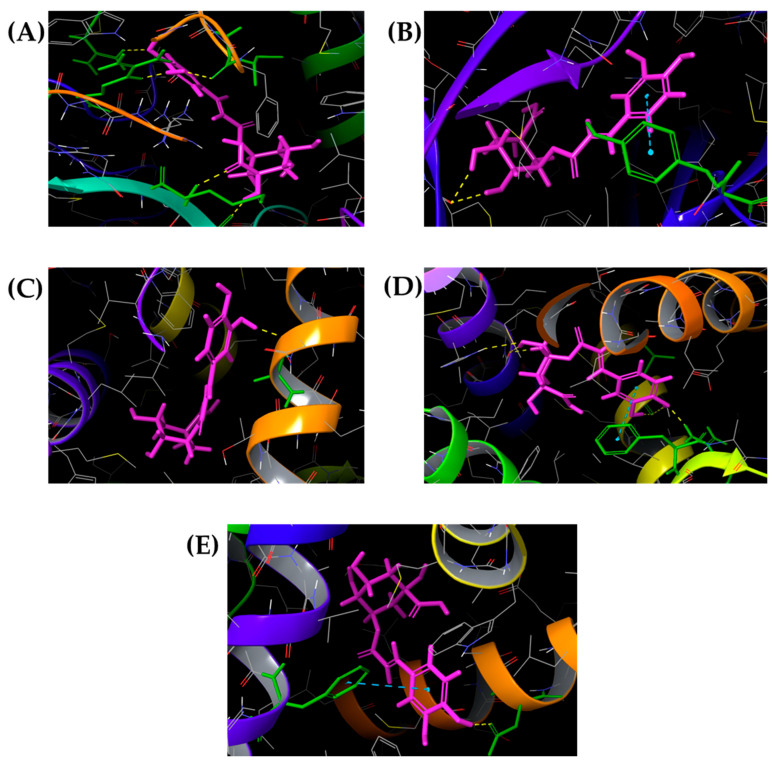
Three-dimensional representation of chlorogenic acid docked with (**A**) aromatase CYP19A1, (**B**) mTOR, (**C**) ERα, (**D**) ERβ, and (**E**) ERRγ. Yellow dashes were used to mark hydrogen bonds while blue dashes are for π-π interaction. Chlorogenic acid is marked in purple color while important amino acid residues are in green.

Docked poses were visualized using the Maestro Schrödinger academic suite (v. 14.4). Hydrogen bonding, π-π stacking, and hydrophobic interactions were analyzed to characterize the stabilization of chlorogenic acid within the ligand binding domain of each target. Hydrogen bonds were depicted as yellow dashed lines, and π-π interactions as blue dashed lines. Three-dimensional binding conformations for chlorogenic acid with the most representative targets are shown in Figure 3 (VEGFR-1, VEGFR-2, CXCR2, nAChR, and TGFBR2) and Figure 4 (CYP19A1, mTOR, ERα, ERβ, and ERRγ).

## 3. Results

### 3.1. Docking Validation and Score Distribution

Re-docking of the co-crystallized ligands reproduced the experimental binding conformations with RMSD ≤ 2.0 Å for all ten receptors, validating the docking protocol. The ChemPLP fitness scores obtained for chlorogenic acid and the corresponding reference ligands are summarized in Table 2 and Table 3. The ChemPLP scores of chlorogenic acid ranged between 57.12 and 69.66, indicating moderate-to-high binding affinity across multiple receptor classes. Among the evaluated targets, the highest docking score was observed for nAChR (69.66), followed by CXCR2 (65.13), ERβ (63.18), TGFBR2 (62.94), while the lowest score corresponded to ERα (57.12) and VEGFR-2 (56.88).

### 3.2. Comparative Binding Affinity

The comparison with co-crystallized ligands revealed that, while the absolute ChemPLP values of chlorogenic acid were lower than those of synthetic inhibitors such as tivozanib (VEGFR-2) and DN200699 (ERRγ), its relative binding efficiency remained notable across multiple targets. The most favorable interaction was observed for CYP19A1 (57.86), consistent with potential aromatase-modulating activity, followed by significant affinities toward mTOR (60.01), ERβ (63.18), and nAChR (69.66). This poly-target binding profile supports the hypothesis that chlorogenic acid can act as ligand for targets involved in angiogenic, hormonal, and signal-transduction pathways, which are highly relevant in CRC biology.

### 3.3. Binding Mode and Interaction Network

A detailed inspection of the best docking poses (Figure 3 and Figure 4) revealed that chlorogenic acid forms multiple polar contacts and π-π stacking interactions within the catalytic or ligand-binding domains of the studied receptors. In fact, the studied polyphenol interacted with VEGFR-1 via hydrogen bonding interactions with Asp1040 and Ile1038 while π-π interactions with Phe1041 contributed to the complex stabilization. On the contrary, chlorogenic acid formed three hydrogen bonds inside the VEGFR-2 ligand binding domain involving Ala1050, Arg1051, and Cys919 residues. Multiple hydrogen bonds between -COOH (Glu249, Lys320) and phenolic -OH groups (Ile73 and Val72) were also responsible for chlorogenic acid binding towards CXCR2. A similar mode of action was observed for nAChR and TGF-β type 2 as well. In CYP19A1, the hydroxyl and carboxyl groups of chlorogenic acid established five hydrogen bonds with Met374, Leu372, Ile133, Arg435, and Arg145. In mTOR, the ligand interacted via two hydrogen bonds with Cys2243, while π-π stacking with Tyr2225 contributed to stabilization in the kinase domain. In nAChR, hydrogen bonds were observed with Ile106, Ile118, Cys190, Glu192, and Tyr195 forming a dense polar network that reinforced ligand retention. For ERα, ERβ, and ERRγ, hydroxyl groups of chlorogenic acid exhibited hydrogen bonding, resembling the interaction patterns reported for other polyphenolic phytoestrogens.

### 3.4. Integrated Structural Insights

The 3D visualizations (Figure 3 and Figure 4) demonstrated that chlorogenic acid consistently occupies hydrophilic and aromatic cavities of its targets, stabilizing the protein–ligand complex through extensive hydrogen bonding and π-π interactions. The polyhydroxylated aromatic framework of the molecule facilitates both electrostatic complementarity and π-π overlap, explaining its broad binding spectrum. Collectively, the results indicated that chlorogenic acid acts as a multi-target ligand (ChemPLP > 60) for proteins involved in angiogenesis (VEGFR-1/2), inflammatory signaling (CXCR2, TGFBR2), hormonal modulation (CYP19A1, ERβ, and ERRγ), and cell growth control (mTOR).

## 4. Discussion

Considering the complexity of the drug discovery process together with associated high costs, long examination period, ethical issues, and the high uncertainty of the outcome, computational drug discovery strategies serve as a “gold standard” in the preliminary examination of the potential therapeutics in the prevention and the treatment of CRC [21]. Regardless of the significant improvements in the detection and the treatment options, this condition is still one of the most common malignant tumors, and a global public health challenge [22]. Phytochemicals, especially polyphenols, are being recognized as safe compounds with pronounced anti-inflammatory and anti-oxidative properties [23]. Specifically, recent studies have illuminated multiple biological functions of chlorogenic acid, highlighting its potential as primary or adjuvant therapy in chronic conditions involving cancer [24,25,26]. Hence, the molecular docking approach was applied in this preliminary study in order to provide binding affinity data and mode of interaction of chlorogenic acid towards ten targets involved in multi-phasic pathogenesis of CRC.

In this context, VEGFR1 and VEGFR2 were examined due to their notable role in the onset and the progression of this cancer. Both receptors are involved in angiogenesis, which is essential for tumor growth and metastasis. Several antiangiogenic agents such as bevacizumab and aflibercept, which targets VEGF, have been on the market for the treatment of metastatic CRC [27]. In addition, chemokines and chemokine receptors such as TGFBR2 and CXCR2 were selected owing to the involvement in the inflammatory processes related to cell growth, differentiation, and immune regulation [16]. A significant expression of TGFBR2 and CXCR2 was found in patients with CRC [16,28]. TGFBR2 behaves as an oncogene and TGFBR2 mutations were documented in up to 40% of patients with CRC, leading to chemotherapy resistance and immunosuppression, and consequently promoting tumor angiogenesis [29,30]. In pre-clinical models, CXCR2 inhibitors reduced tumor growth as well as vascular development [31]. The inhibition of TGFBR2 had an important role in CRC treatment via a Wnt pathway that regulated β-catenin activation, which had a key role in CRC progression [30,32]. Targeting mTOR as part of the PI3K/AKT/mTOR pathway was also critical in addressing CRC due to its role in cell proliferation and survival [33]. This signaling pathway is recognized as a major resistance mechanism to CRC therapy. Until now, several drugs classified as mTOR inhibitors have been developed and the potential of rapamycin in CRC was investigated in clinical trials [34]. Interestingly, Rhein, a natural anthraquinone compound, showed promising results as an mTOR inhibitor in pre-clinical trials leading to the decrease in VEGF expression in CRC cells [35]. Therefore, understanding the potential of chlorogenic acid as an mTOR inhibitor can provide a rationale for combination and patient-targeted therapies in CRC. NAChR, widely distributed in the human body, was tested as a potential target involved in migration, proliferation, and invasion of tumor cells based on recent reports [36]. Although the underlying mechanisms related to this receptor have not been fully elucidated in patients with CRC, nACh behaves as an autocrine or paracrine hormone. The activation of this receptor suppresses the inflammation and consequently tumorigenesis while its activation in tumor cells triggers the cell proliferation and migration. Hence, owing to the anti-inflammatory properties of vagal signaling, the designing of novel pharmacological agents that targets this receptor might have beneficial effects in CRC [37,38]. Moreover, considering the controversial estrogenic role in the pathogenesis of multiple cancers, especially CRC [17], chlorogenic acid affinity towards CYP19A1 together with estrogen receptors (ERα, ERβ, and ERRγ) were investigated as well. Although both ERα and ERβ are expressed in healthy colorectal tissue, in CRC, downregulation of ERβ and overexpression of ERα has been observed [39]. In addition, ERβ expressed a protective role in CRC through promoted reduction in cancer cell growth in clinical colon samples. ERRγ has been reported as a potential suppressor of CRC aggressiveness by controlling the Wnt/β-catenin pathway. CYP19A1, as a key enzyme in estrogen biosynthesis, was also considered as a potential target due to its role in the regulation of chemoresistance in CRC. Hence, the binding of a plant-derived compound to ERs and ERRγ might have beneficial effects in CRC prevention [17,39,40,41].

Based on the obtained binding affinities and modes of interactions, hydroxyl, carboxyl, and phenolic -OH groups were identified as crucial for chlorogenic acid’s multi-target properties (Figure 3 and Figure 4). These structural features had a key role in its bioactivity, attributed to chronic conditions associated with induced inflammation and oxidative stress in the gut mucosa [42]. As the most abundant intermolecular force in the biological systems, hydrogen bonding plays a major role in protein–ligand binding affinity and selectivity [43]. In addition, the benzene ring of chlorogenic acid was involved in π-π interactions with selected targets and, in particular, VEGFR1, mTOR, ERβ, and ERRγ, and contributed to the obtained ChemPLP score (Table 2 and Table 3). The stability of a low molecular weight ligand–protein complex was further increased by the noncovalent attractive forces between the aromatic rings of chlorogenic acid and corresponding Phe residues [44].

The drug-likeness of chlorogenic acid was comparable to that of tivozanib, the only approved VEGFR-targeted treatment. In fact, chlorogenic acid formed hydrogen bonds with the key active site’s residues Cys919 and Asp1040 [45]. Despite limited knowledge regarding the amino acid residues involved in the activation of human CXCR2 receptor, chlorogenic acid established a network of interactions with surrounding amino acids, suggesting its potential to modulate CXCR2 activation and G-protein coupling (Figure 3C). Ligand-binding analysis revealed that chlorogenic acid formed dense hydrogen bonds with active site residues of nAChR (Figure 3D). Residues Ile106, Ile118, and Tyr195 were also identified as important contributors for the robust inhibitory properties previously reported for phytochemical derivatives targeting nAChR [46]. Promising activity towards TGFBR2 could also be expected for chlorogenic acid, owing to its good stability inside the active site. Multiple hydrogen bonds were formed between the hydroxyl, carboxyl, and phenolic hydroxyl groups of chlorogenic acid and amino acid residues (Figure 3E). As shown in Figure 4B, the complex formed between chlorogenic acid and mTOR was stabilized via the combination of a hydrogen bonds between hydroxyl groups and Cys2243, as well as π-π interactions with Tyr2225. The similar mode of interaction inside TGFBR2 and the mTOR ligand binding domain was observed for novel benzenesulfonamide–thiazolidinone derivatives [47].

Regarding the aromatase CYP19A1 role in the progression of estrogen-related tumors, chlorogenic acid was also validated as a potential enzyme inhibitor. Molecular docking results showed high binding affinity through key hydrogen bonding with Met374, Leu372, and Ile133 involving carbonyl oxygen, the hydroxyl group, and phenolic -OH groups of chlorogenic acid (Table 3, Figure 4A). These results are in agreement with the novel complementary in silico data that attribute the important role of those amino acids in the aromatase CYP19A1 inhibition [48]. In addition, the docking pose of chlorogenic acid that corresponded to the highest ChemPLP score exhibited hydrogen bonding interactions inside the active site of the receptor ERα via phenolic -OH groups which agrees with the ERα binder’s features [49]. It is worth noting that chlorogenic acid reproduced the 17-β estradiol mode of action, making the network of hydrogen and hydrophobic interactions inside the ERβ ligand binding domain (His475, Leu339, and Phe356). Moreover, similar with resveratrol, chlorogenic acid established the additional hydrogen bond with Arg346 (Figure 4D) [50]. Being a part of the estrogen-related receptor family, ERRγ could be found in tissues with the increased metabolic demand [51]. Hence, the affinity of chlorogenic acid to interact with this target was also studied. Asp273 was involved in H-bonding while Phe435 contributed to the additional stabilization through hydrophobic π-π interactions. These findings are in accordance with the behavior of some estrogen-like chemicals [51].

Bearing in mind the good stability governed by the combination of hydrogen and π-π stacking interactions inside catalytic or ligand-binding domains of the tested receptors/enzymes, promising multi-target activity of chlorogenic acid might be expected in vitro.

Furthermore, experimental evidence has indicated that chlorogenic acid reduces CRC cell viability in a dose-dependent manner and induced cell cycle arrest in HT-29 cells through the upregulation of p21 and p53 [52]. Concomitantly, chlorogenic acid promoted apoptotic signaling by downregulating Bcl-2 and NF-κB while enhancing caspase-3 and caspase-9 activation and increasing intracellular reactive oxygen species levels. Taken together, these findings demonstrated that chlorogenic acid exerts cytotoxic and pro-apoptotic effects in CRC cells under in vitro conditions [53]. Although these results did not establish therapeutic efficacy, they provided biologically plausible experimental support for further mechanistic and translational studies investigating chlorogenic acid in CRC research. In a chemically induced mouse model of colon carcinogenesis, the administration of caffeine and chlorogenic acid, either alone or in combination, demonstrated modulatory effects on early tumorigenic events. Notably, the combined treatment exerted the most pronounced biological impact, significantly reducing epithelial cell proliferation and enhancing apoptosis within colonic crypts. This effect was accompanied by a marked decrease in pro-inflammatory cytokines and downregulation of the oncomiR miR-21a-5p, with consequent modulation of target genes involved in the regulation of proliferation, inflammation, and apoptosis. Collectively, these findings indicated that the combination of coffee-derived compounds can attenuate early-stage colon carcinogenesis in an experimental setting. Despite the lack of direct clinical efficacy, these findings provided mechanistic in vivo support for a potential chemopreventive role of chlorogenic acid, highlighting the relevance of dietary factors in CRC prevention and warranting further translational investigation [54].

From a clinical standpoint, chlorogenic acid could represent a promising complementary agent in CRC management. Its multi-target molecular profile, spanning angiogenesis, inflammation, metabolic regulation, and hormonal pathways, suggests a pleiotropic mechanism potentially capable of modulating both tumor progression and microenvironmental inflammation. These pharmacodynamic features are consistent with the emerging concept of polypharmacology in oncology, whereby agents acting on multiple interconnected pathways may enhance therapeutic efficacy and reduce resistance development [55]. Indeed, pre-clinical models have already indicated that chlorogenic acid may suppress tumor growth, reduce metastatic potential, and enhance the sensitivity of malignant cells to conventional chemotherapeutic drugs such as 5-fluorouracil [56,57]. Moreover, its favorable safety profile, oral bioavailability, and ability to attenuate oxidative stress and intestinal mucosal injury could provide added value in adjuvant treatment settings [26]. However, translation into clinical practice still requires well-controlled trials to define effective dosing, pharmacokinetic profile, and potential interactions with standard oncologic regimens. Given its natural origin and dual antioxidant and anti-inflammatory action, chlorogenic acid may also be explored as part of dietary or nutraceutical interventions aimed at CRC prevention, especially in high-risk populations [58]. Further in vitro and in vivo studies integrating metabolomic and pharmacogenomic profiling will be crucial to validate these preliminary computational findings and to elucidate its clinical potential as a safe, multi-target therapeutic candidate in CRC.

## 5. Conclusions

This preliminary in silico study has all the strengths and limitations of molecular docking analysis. The obtained results suggest that chlorogenic acid may interact with multiple molecular targets implicated in CRC-related pathways, including those involved in angiogenesis, inflammation, and tumor progression. In addition, visualization of the best ligand-target poses enabled the prediction of ligand-target interactions at a molecular level. The docking analyses indicated favorable interaction patterns, with hydroxyl and phenolic groups contributing to hydrogen bonding and π-π stacking interactions. Although these results provide in silico insight into the potential multi-target interaction profile of chlorogenic acid, they should be interpreted with caution. The major method limitations include the application of scoring functions and restricted flexibility of receptor conformations in pose prediction. Indeed, the in silico approach based on static docking scores could not establish biological effects, affinity, selectivity and functional modulation. However, this analysis enables the identification of promising compounds of therapeutic interests as well as the understanding of the relationships between different molecular targets involved in CRC. Given the potential offered by the obtained results, molecular dynamics simulations should be directed towards the investigation of the pose stability and binding free energy considering target flexibility and solvent effects. At the end, future in vitro and in vivo studies are necessary to confirm target engagement, evaluate pharmacokinetic and bioavailability constraints, and determine whether the predicted interactions translate into meaningful biological effects. Within these limitations, the present work contributes to hypothesis generation and may inform subsequent experimental research on chlorogenic acid in CRC.

## Figures and Tables

**Table 1:** Target used in docking analyses and their PDB entry codes.

Target	PDB Entry Code	Redocked Co-Crystalized Ligand
VEGFR-1	3HNG	*N*-(4-Chlorophenyl)-2-((pyridin-4-ylmethyl)amino)benzamide
VEGFR-2	4ASE	Tivozanib
CXCR2	6LFL	4-[[3,4-bis(oxidanylidene)-2-[[(1~{R})-1-(4-propan-2-ylfuran-2-yl)propyl]amino]cyclobuten-1-yl]amino]-~{*N*},~{*N*}-dimethyl-3-oxidanyl-pyridine-2-carboxamide
nAChR	4ZK4	(5R)-1-methyl-7-[5-(propan-2-yloxy)pyridin-3-yl]-1,7-diazaspiro[4.4]nonane
TGFBR2	5QIN	*N*-{4-[3-(6-methoxypyridin-3-yl)-1*H*-pyrrolo[3,2-b]pyridin-2-yl]pyridin-2-yl}acetamide
Aromatase CYP19A1	5JL6	4-androstene-3-17-dione
mTOR	4JT5	2-[4-amino-1-(propan-2-yl)-1*H*-pyrazolo[3,4-d]pyrimidin-3-yl]-1*H*-indol-5-ol
ERα	7UJO	(9beta,11beta,17beta)-11-{4-[2-(dimethylamino)ethoxy]phenyl}estra-1,3,5(10)-triene-3,17-diol
ERβ	5TOA	Estradiol
ERRγ	6KNR	DN200699

Abbreviations: VEGFR-1, Vascular Endothelial Growth Factor Receptor 1; VEGFR-2, Vascular Endothelial Growth Factor Receptor 2; CXCR2, C-X-C Motif Chemokine Receptor 2; nAChR, Nicotinic Acetylcholine Receptor; TGFBR2, Transforming Growth Factor Beta Receptor Type II; CYP19A1, Cytochrome P450 Aromatase; mTOR, Mechanistic Target of Rapamycin; ERα, Estrogen Receptor Alpha; ERβ, Estrogen Receptor Beta; ERRγ, Estrogen-Related Receptor Gamma.

**Table 2:** The best ChemPLP scores obtained for chlorogenic acid towards VEGFR-1, VEGFR-2, CXCR2, nAChR, and TGFBR2 in comparison to co-crystalized ligands.

Compound	VEGFR-1	VEGFR-2	CXCR2	nAChR	TGFBR2
Chlorogenic acid	60.5381	56.8755	65.1313	69.6624	62.9376
*N*-(4-Chlorophenyl)-2-((pyridin-4-ylmethyl)amino)benzamide	97.1528	/	/	/	/
Tivozanib	/	106.7204	/	/	/
4-[[3,4-bis(oxidanylidene)-2-[[(1~{R})-1-(4-propan-2-ylfuran-2-yl)propyl]amino]cyclobuten-1-yl]amino]-~{*N*},~{*N*}-dimethyl-3-oxidanyl-pyridine-2-carboxamide	/	/	103.3357	/	/
(5R)-1-methyl-7-[5-(propan-2-yloxy)pyridin-3-yl]-1,7-diazaspiro[4.4]nonane	/	/	/	81.2601	/
*N*-{4-[3-(6-methoxypyridin-3-yl)-1*H*-pyrrolo[3,2-b]pyridin-2-yl]pyridin-2-yl}acetamide	/	/	/	/	92.0903

Abbreviations: VEGFR-1, Vascular Endothelial Growth Factor Receptor 1; VEGFR-2, Vascular Endothelial Growth Factor Receptor 2; CXCR2, C-X-C Motif Chemokine Receptor 2; nAChR, Nicotinic Acetylcholine Receptor; TGFBR2, Transforming Growth Factor Beta Receptor Type II.

**Table 3:** The best ChemPLP scores obtained for chlorogenic acid towards aromatase CYP19A1, ERα, ERβ, and ERRγ in comparison to co-crystalized ligands.

Compound	Aromatase CYP19A1	mTOR	ERα	ERβ	ERRγ
Chlorogenic acid	57.8643	60.0097	57.1267	63.1841	60.8226
4-androstene-3-17-dione	60.6403	/	/	/	/
2-[4-amino-1-(propan-2-yl)-1*H*-pyrazolo[3,4-d]pyrimidin-3-yl]-1*H*-indol-5-ol	/	75.859	/	/	/
(9beta,11beta,17beta)-11-{4-[2-(dimethylamino)ethoxy]phenyl}estra-1,3,5(10)-triene-3,17-diol	/	/	100.3033	/	/
Estradiol	/	/	/	74.9266	/
DN200699	/	/	/	/	118.9592

Abbreviations: CYP19A1, Cytochrome P450 Aromatase; mTOR, Mechanistic Target of Rapamycin; ERα, Estrogen Receptor Alpha; ERβ, Estrogen Receptor Beta; ERRγ, Estrogen-Related Receptor Gamma.

## Data Availability

Data are contained within the article.

## Abbreviations

The following abbreviations are used in this manuscript:

2D	Two-dimensional
3D	Three-dimensional
ChemPLP	Chemical piecewise linear potential
CRC	Colorectal cancer
CXCR2	C-X-C motif chemokine receptor 2
CYP19A1	Cytochrome P450 aromatase
ERα	Estrogen receptor alpha
ERβ	Estrogen receptor beta
ERRγ	Estrogen-related receptor gamma
GOLD	Genetic Optimization for Ligand Docking
MMFF94	Merck Molecular Force Field 94
mTOR	Mechanistic target of rapamycin
nAChR	Nicotinic acetylcholine receptor
PDB	Protein Data Bank
PGI	Protected geographical indication
PI3K/AKT/mTOR	Phosphatidylinositol-3-kinase/Protein kinase B/Mechanistic target of rapamycin pathway
RMSD	Root-mean-square deviation
TGFBR2	Transforming growth factor-β receptor type 2
VEGFR-1	Vascular endothelial growth factor receptor 1
VEGFR-2	Vascular endothelial growth factor receptor 2
